# Serum PFOA Levels in Residents of Communities Near a Teflon-Production Facility

**DOI:** 10.1289/ehp.10468

**Published:** 2007-10

**Authors:** Myra L. Karstadt

**Affiliations:** Drexel University School of Public Health, Philadelphia, Pennsylvania, E-mail: myrakarstadt@verizon.net

In a recent article, Tillett (2007) reported on research by the University of Pennsylvania NIEHS (National Institute of Environmental Health Sciences) Center of Excellence in Environmental Toxicology (CEET). CEET deputy director Edward Emmett described analyses of perfluorooctaonic acid (PFOA) in blood serum collected during mid-2004 from residents of towns near DuPont’s Teflon production facility in Parkersburg, West Virginia. Results of the analyses, which identified PFOA levels “60–75 times higher than in the general population,” were presented at a community meeting in October 2005, and “[DuPont] began offering bottled water to all residents being serviced in the Little Hocking Water District within days” of the meeting (Tillett 2007).

If the criterion for offering bottled water was high serum PFOA levels in residents of West Virginia and Ohio towns with PFOA-contaminated drinking water, then DuPont should have offered bottled water to Little Hocking, Ohio, and other towns well before October 2005. There would have been ample reason for concern about high serum PFOA levels because by 2001 there were reports in the scientific literature of animal studies that showed PFOA to be a developmental and liver toxicant, as well as a multisite carcinogen (Morgan and Cory-Slechta 2006).

In July 2004, the U.S. Environmental Protection Agency (EPA) sued DuPont for failure to file with the agency reports on PFOA required to be submitted under section 8(e) of the Toxic Substances Control Act [TSCA 8(e)] (U.S. EPA 2004a). In December 2004, a count was added (U.S. EPA 2004b) covering the company’s failure to submit data obtained in July 2004 indicating that 10 community residents exposed to PFOA-contaminated drinking water in the area near Parkersburg, West Virginia, had serum levels of PFOA ranging from 15.7 to 128 ppb (mean, 67 ppb) (U.S. EPA 2004b) The U.S. average serum level of PFOA is approximately 5 ppb (U.S. EPA 2004b).

Each of the 10 individuals in the 2004 group was exposed to PFOA through drinking water provided by the Lubeck (WV) Public Service District (LPSD) where, according to DuPont,

[the level of PFOA in the drinking water] averaged approximately 0.5 ppb over the last several years. All ten of the individuals tested claim to have stopped using the contaminated public drinking water as their primary source of drinking water approximately three years ago. (Bilott 2004)

The concentration of PFOA in LPSD water (0.5 ppb) was well below recent levels in Little Hocking, but serum PFOA levels were still several times greater than 5 ppb. Also, given the half-life of PFOA in humans of approximately 4 years, it is likely that the high serum levels in the test group reflected exposures through drinking PFOA-contaminated water > 3 years before serum was taken, plus ongoing exposure from kitchen use, bathing/showering, and home-grown fruits and vegetables. The possible importance of exposure from home-grown fruits and vegetables was noted for the Little Hocking population (Tillett 2007).

Included in the U.S. EPA’s December 2005 settlement of the agency’s case against DuPont, although not mentioned in the agency press release (U.S. EPA 2005a), was a count related to DuPont’s failure to submit to the U.S. EPA, as required by TSCA 8(e), the results of analyses of serum samples taken in 2002 from the same 10 people whose serum was analyzed in 2004 (U.S. EPA 2005b). PFOA levels in the 2002 survey were reported as 10–85 ppb (mean, 33.3 ppb). The 2002 data were submitted to the U.S. EPA in December 2004 (Robertson 2005) by Robert Bilott, the principal attorney in a civil suit against DuPont for contaminating drinking water with PFOA in the Ohio River Valley.

Also, 5 of the 10 individuals in the 2002 and 2004 studies participated in a 2001 PFOA analysis (Robertson 2005). DuPont disclaimed participation in or substantive knowledge of that survey, and the 2001 data were not included in the U.S. EPA’s settlement with DuPont. In 2001, PFOA levels in the five survey participants ranged from 13 to 63 ppb (mean, 37.2 ppb).

It would be helpful if members of the public had ready access to the results of the 2001, 2002, and 2004 LPSD population surveys. A bibliographic database [Toxic Substances Control Act Test Submissions (TSCATS), U.S. EPA, Washington, DC] tracked TSCA 8(e) submissions to the U.S. EPA and could be accessed through such data providers as TOXLINE (National Library of Medicine 2007), but TSCATS was shut down some years ago; it appears that, regarding TSCA 8(e) reports on PFOA, TOXLINE is current only through perhaps 2001 at the latest (National Library of Medicine 2007). The majority of TSCA 8(e) reports on PFOA were submitted to the agency starting in 2001. PDF versions of TSCA 8(e) reports for approximately 2000–2006 are nominally available through a U.S. EPA website (U.S. EPA 2007); as of April 2007, submissions for 2000–2004, which had been online 2 years ago, were no longer available (U.S. EPA 2007). The 2004 survey data are available online at that U.S. EPA website (Bilott 2004).

## Editor’s note:

We appreciate Karstadt’s time and interest in commenting on our news article, and her added perspective on the circumstances surrounding the Little Hocking and Parkersburg exposures.
